# Pain and sedation management and monitoring in pediatric intensive care units across Europe: an ESPNIC survey

**DOI:** 10.1186/s13054-022-03957-7

**Published:** 2022-03-31

**Authors:** Daverio Marco, Florian von Borell, Anne-Sylvie Ramelet, Francesca Sperotto, Paula Pokorna, Sebastian Brenner, Maria Cristina Mondardini, Dick Tibboel, Angela Amigoni, Erwin Ista, the Analgosedation CONSORTIUM on behalf of the Pharmacology Section and the Nurse Science Section of the European Society of Paediatric and Neonatal Intensive Care, Ermira Kola, Maria Vittinghoff, Elim Duval, Branka Polić, Frédéric Valla, Felix Neunhoeffer, Tziouvas Konstantinos, Zoltán Györgyi, Mong Hoi Tan, Antigona Hasani, Edita Poluzioroviene, Reinis Balmaks, Mickael Afanetti, Gunnar Bentsen, Alicja Bartkowska-Sniatkowska, Cristina Camilo, Dusica Simic, Yolanda M. López-Fernández, Janet Mattsson, Hasan Özen, Dmytro Dmytriiev, Joseph C. Manning, Hakan Tekgüç

**Affiliations:** 1 Pediatric Intensive Care Unit, Department of Woman’s and Child’s Health, University Hospital of Padua, Padua, Italy; 2 Department of Pediatric Cardiology and Intensive Care Medicine, Hannover Medical School, Hannover, Germany; 3 Institute of Higher Education and Research in Healthcare, Faculty of Biology and Medicine, University of Lausanne, Lausanne, Switzerland; 4 Department Woman-Mother-Child, Lausanne University Hospital, Lausanne, Switzerland; 5 Department of Cardiology, Boston Children’s Hospital, Harvard Medical School, Boston, MA USA; 6 Institute of Pharmacology, First Faculty of Medicine, Charles University and General University Hospital, Prague, Czech Republic; 7 Department of Paediatrics and Inherited Metabolic Disorders, First Faculty of Medicine, Charles University and General University Hospital, Prague, Czech Republic; 8 Intensive Care and Department of Paediatric Surgery, Erasmus Medical Center Sophia Children’s Hospital, Rotterdam, The Netherlands; 9 Department of Physiology and Pharmacology, Karolinska Institutet and Karolinska University Hospital, Stockholm, Sweden; 10 Department of Pediatric and Adolescent Medicine, University Clinic Carl Gustav Carus, Dresden, Germany; 11 Pediatric Anesthesia and Intensive Care Unit, Department of Woman’s and Child’s Health, IRCCS University Hospital of Bologna Policlinico S.Orsola, Bologna, Italy; 12 Department of Woman’s and Child’s Health, IRCCS University Hospital of Bologna Policlinico S.Orsola, Bologna, Italy; 13 Pediatric Intensive Care Unit, Department of Pediatric Surgery, • Erasmus MC – Sophia Children’s Hospital, University Medical Center Rotterdam, Rotterdam, The Netherlands; 14 University Hospital Centre, Tirana, Albania; 15 Department of Anaesthesiology and Intensive Care Medicine, Medical University, Graz, Austria; 16 Antwerp University Hospital, Antwerp Edegem, Belgium; 17 Paediatric Intensive Care Unit Department of Paediatrics, University Hospital of Split, Split, Croatia; 18 Paediatric Intensive Care Hôpital Femme Mère Enfant, Hospices Civils de Lyon, Lyon, France; 19 Department of Pediatric Cardiology, Pulmonology and Intensive Care Medicine, University Children’s Hospital, Tübingen, Germany; 20 Paediatric Intensive Care Unit Aglaia and Panagiotis, Kyriakou Children’s Hospital, Athens, Greece; 21 2nd Department of Pediatrics, Semmelweis University, Budapest, Hungary; 22 Pediatric Intensive Care Unit, Children’s Health Ireland, Crumlin, Ireland; 23 Department of Anesthesiology, Reanimation and Emergency Medicine Faculty of Medicine, University of Prishtina, Prishtina, Kosovo Serbia; 24 Vilnius University Hospital Santaros Klinikos, Vilnius, Lithuania; 25 Intensive Care Unit, Children’s Clinical University Hospital, Riga, Latvia; 26 Pediatric Intensive Care Unit, Hôpitaux Pédiatriques de Nice, Nice, France; 27 Department of Anaesthesiology, Oslo University Hospital, Rikshospitalet, Norway; 28 Department of Paediatric Anaesthesiology and Intensive Therapy, Poznan University of Medical Sciences, Poznan, Poland; 29 Paediatric Intensive Care Unit, Department of Paediatrics, Hospital de Santa Maria - CHULN, Lisbon, Portugal; 30 University Children’s Hospital Medical Faculty, University of Belgrade, Belgrade, Serbia; 31 Cruces University Hospital Biocruces-Bizkaia Health Research Institute, Basque Country, Spain; 32 Intensive Care Red Cross University, Stockholm, Sweden; 33 Department of Pediatric Critical Care Medicine, Ankara University Faculty of Medicine, Ankara, Turkey; 34 Department of Anesthesiology and Intensive Care, Vinnitsa National Medical University, Vinnytsia Oblast, Ukraine; 35 Nottingham Children’s Hospital, University of Nottingham, Nottingham, UK; 36 Paediatric Intensive Care Unit, Department of Pediatrics, Dr. Burhan Nalbantoglu State Hospital, Lefkosa, North Cyprus Turkey

**Keywords:** Analgesia, Sedation, Critical care, Pediatric intensive care unit, Monitoring

## Abstract

**Background:**

Management and monitoring of pain and sedation to reduce discomfort as well as side effects, such as over- and under-sedation, withdrawal syndrome and delirium, is an integral part of pediatric intensive care practice. However, the current state of management and monitoring of analgosedation across European pediatric intensive care units (PICUs) remains unknown. The aim of this survey was to describe current practices across European PICUs regarding the management and monitoring of pain and sedation.

**Methods:**

An online survey was distributed among 357 European PICUs assessing demographic features, drug choices and dosing, as well as usage of instruments for monitoring pain and sedation. We also compared low- and high-volume PICUs practices. Responses were collected from January to April 2021.

**Results:**

A total of 215 (60% response rate) PICUs from 27 European countries responded. Seventy-one percent of PICUs stated to use protocols for analgosedation management, more frequently in high-volume PICUs (77% vs 63%, *p* = 0.028). First-choice drug combination was an opioid with a benzodiazepine, namely fentanyl (51%) and midazolam (71%) being the preferred drugs. The starting doses differed between PICUs from 0.1 to 5 mcg/kg/h for fentanyl, and 0.01 to 0.5 mg/kg/h for midazolam. Daily assessment and documentation for pain (81%) and sedation (87%) was reported by most of the PICUs, using the preferred validated FLACC scale (54%) and the COMFORT Behavioural scale (48%), respectively. Both analgesia and sedation were mainly monitored by nurses (92% and 84%, respectively). Eighty-six percent of the responding PICUs stated to use neuromuscular blocking agents in some scenarios. Monitoring of paralysed patients was preferably done by observation of vital signs with electronic devices support.

**Conclusions:**

This survey provides an overview of current analgosedation practices among European PICUs. Drugs of choice, dosing and assessment strategies were shown to differ widely. Further research and development of evidence-based guidelines for optimal drug dosing and analgosedation assessment are needed.

**Supplementary Information:**

The online version contains supplementary material available at 10.1186/s13054-022-03957-7.

## Keypoints

- *Question* What is the pain and sedation management and monitoring practices in pediatric intensive care units (PICUs) across Europe?
- *Findings* In this survey supported by ESPNIC, which included 215 European PICUs, we described a significant variation in practice, either in the use of protocols for analgosedation, or the type and dose of the drugs of choice, or the type and frequency of analgosedation monitoring.
- *Meaning* Pain and sedation management and monitoring in European PICUs varies widely. New research and evidence-based guidelines on the topic that reflect European practices are needed.

## Introduction

Management of pain and sedation is an integral part of the pediatric intensive care practice [1]. Providing pain relief and sedation to ensure optimal comfort and avoid complications is a challenging balancing act for healthcare providers. The difficulties are related to a wide range of developmental ages in critically ill children, the inability to communicate, the complexity of the clinical status, and the highly variable pharmacodynamics and pharmacokinetics metabolisms [2]. The goal is to keep the child pain-free but sufficiently awake for optimal recovery. If undersedation can result in unnecessary psychological and physical stress as well as accidental extubation, oversedation can lead to prolonged mechanical ventilation and pediatric intensive care unit (PICU) length of stay, iatrogenic withdrawal syndrome, and delirium [3, 4].

To date, only a few international and national clinical practice guidelines for the management of pain and sedation in children are available [5–8]. Except for the recently published PANDEM guideline, these guidelines were published more than 10 years ago with recommendations based on limited evidence. None of them addressed the intensive care neonatal population. Other guidelines focused only on the recognition and assessment of pain in children with no mention regarding its management [9]. The position statement of experts from the European Society of Paediatric and Neonatal Intensive Care (ESPNIC) was developed to guide assessment of pain, sedation, delirium, and withdrawal in PICU using appropriate tools [10]. A recent systematic review also provides a useful list of pain and sedation scales for preverbal children, enforcing the important message to use a validated instrument for the targeted population and type of pain of interest [11].

Previous surveys developed in different countries regarding the utilization of pain and sedation tools in PICUs have shown wide variation in their availability and application into standardized care practices [12–14]. However, data on the assessment and management of pain and sedation across Europe are currently missing. The main objective of this survey was to describe the analgesia and sedation monitoring and management practices in the different PICUs in Europe. Secondary objectives were to compare practices between high- and low-volume PICUs.

## Methods

### Study design

We conducted a cross-sectional anonymous electronic survey focused on the evaluation of analgesia and sedation practices and monitoring across European PICUs.

### Survey development and testing

The survey instrument was developed in English and formatted using the web-based Google Forms software (https://www.google.com/forms/about/). The survey was designed to address all aspects of the research question. The question domains and specific questions were built on an extensive review of the literature and experiential multidisciplinary knowledge of the pain and sedation practice. The survey was developed by the authors belonging to the Pharmacology section of the European Society of Paediatric and Neonatal Intensive Care (ESPNIC) (AA, DT, MCM, MD, FS, PP) and reviewed by the authors from the ESPNIC Nursing Science Section (ASR, EI) to reflect the multidisciplinary nature of analgesia and sedation practice in the PICU.

Subsequently, the survey was pilot tested with 10 pediatric intensivists for clarity and face validity [15]. The survey consisted of 56 questions divided into three sections, and required 10–15 min on average to be completed (see Additional file 1: Appendix): Part A. PICU and patients characteristics, including location, type and size of the PICU; Part B. Analgesia and sedation practice, including specific information on the drugs used as first and second choices, minimum and maximum drug doses, and use of neuromuscular blocking agents (NMBA); Part C. Analgesia and sedation assessment and monitoring. We used single- and multiple-choice questions, closed-ended and free text questions to allow for comprehensive detailed information on each topic, and facilitate data analyses and comparisons.

### Recruitment of European PICUs and data collection

This survey targeted intensivists and nurses working in PICUs (i.e. PICUs, mixed neonatal and pediatric ICUs, mixed adult and pediatric ICUs) in Europe. Using ESPNIC and personal networks, one representative for each European country (named from now forward as “country-lead”) was contacted in January 2021 and considered responsible for disseminating the survey by contacting one PICU referent for each PICU in their own country. We recommended each country-lead to contact only one referent for each PICU in order to avoid duplicates. To maximize the response rate, reminders were initially sent to all country-leads and were subsequently targeted to country-leads with a low response rate, only. No identifiable staff and patient data were collected, and consent was implied by completing the survey. The survey diffusion started on 26 January 2021. All valid responses received before 16 April 2021 were included in the analysis.

### Data analysis

Raw data downloaded from Google form were checked for data completeness and potential duplicates, which were removed keeping the first response from each unit. Data were analysed using STATA (version 17.0, StataCorp LP, College Station, TX, USA). Descriptive data were reported as frequency (proportion) for categorical variables, and median (interquartile ranges, IQR) for continuous variables given their nonparametric distribution. Analgesia and sedation characteristics were subsequently compared between two groups of PICUs, based on the PICU yearly admission volume: specifically, PICUs with more than 450 admissions per year (“high-volume PICUs”) were compared to PICUs with less or equal to 450 admissions per year (“low-volume PICUs”). Since there is no consensus definition in the literature for “high-” or “low-volume” PICUs, we used the median number of admissions per PICUs, calculated in our survey, as a threshold to define these two groups. The Wilcoxon sum-rank test was used to compare continuous variables, and the Pearson Chi-square test, or the Fisher-exact test when appropriate (*n* < 5 in > 20% cells), was used for comparison of categorical variables.

## Results

### Survey responders

**Fig. 1:**
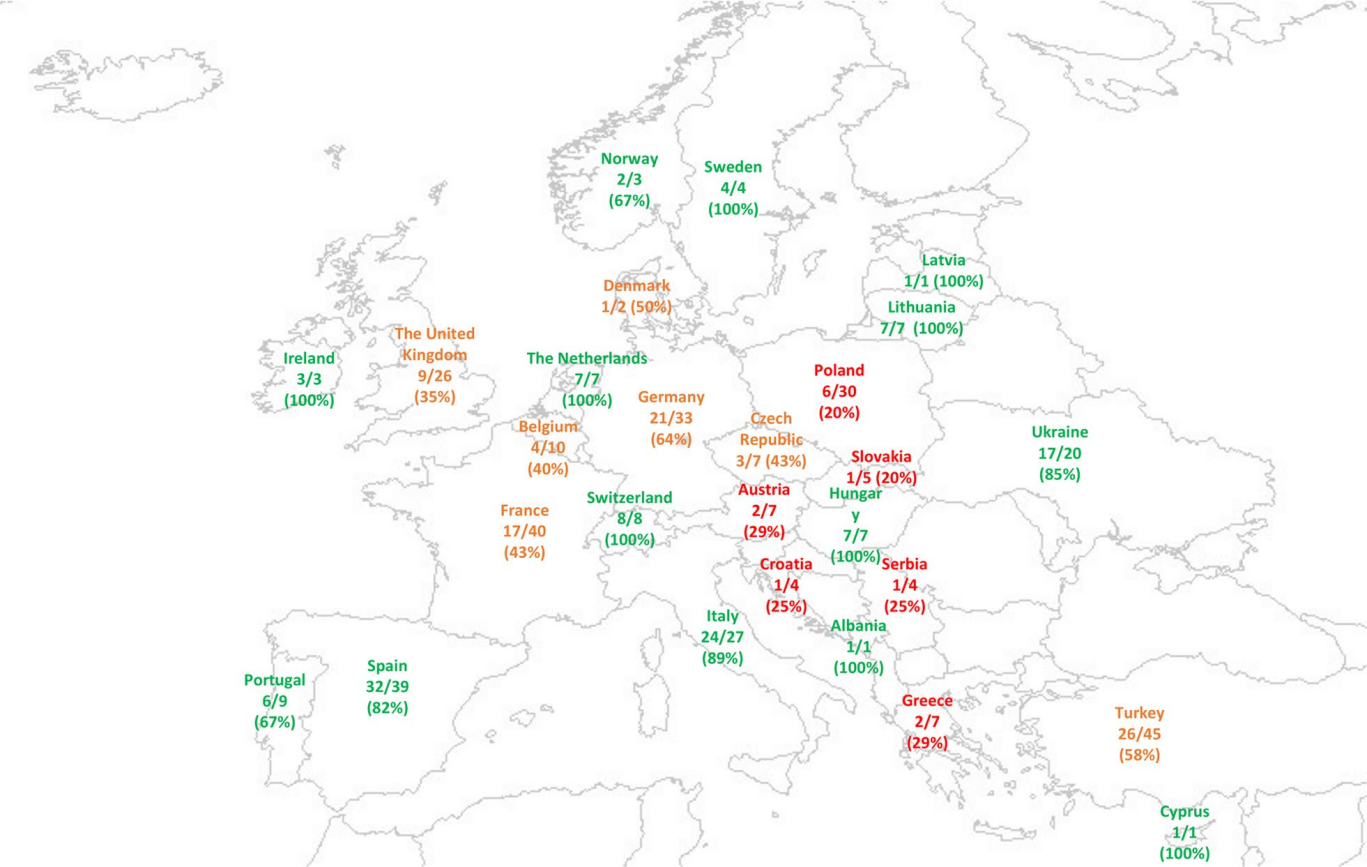
Map describing the distribution of survey responders across European countries. The number of PICUs who replied to the survey (numerator) is reported along with the number of PICUs in each country (denominator) and the percentage of responders. In red countries with a response rate < 33%, in orange 33–66% and green > 66%

Out of the 38 contacted country-leads, 27 responded. A total of 357 PICU representatives received the invitation to participate in the survey and 224 completed the survey. After excluding eight duplicates and one ICU admitting neonates only, the total number of responders was 215 (60% response rate) from 27 countries, with a response rate per country ranging from 20 to 100% (Fig. 1). The vast majority of the responders were from academic/teaching hospitals (196, 91%). Most of the responders were pediatricians (139, 65%), while a minority were nurses (20, 9%) or surgeons (4, 2%) (Table 1).

**Table 1:** PICUs, responders, and protocol characteristics according to yearly admission volume

Characteristics	Total PICUs *n* = 215	PICUs ≤ 450 admissions/year *n* = 117	PICUs > 450 admissions/year *n* = 98	*p* value
Type of PICU				
Pediatric ICU	158 (74)	77 (66)	81 (83)	0.003
Mixed neonatal and pediatric ICU	48 (22)	27 (31)	17 (17)	
Mixed adult and pediatric ICU	9 (4)	9 (8)	0 (0)	
PICU in a teaching/academic hospital	196 (91)	111 (95)	85 (87)	0.036
PICU admitting post-cardiac surgery	69 (32)	27 (23)	42 (43)	0.002
PICU providing palliative care/terminal sedation	182 (85)	101 (87)	81 (83)	0.457
PICUs dimensions				
Maximum bed capacity, min–max	9 (7–16), 2–35	8 (6–10), 2–27	14 (9–18), 6–35	–
Number of admissions per year, min–max	450 (260–700), 30–2050	300 (200–400), 30–450	700 (560–980), 460–2050	–
Responders’ role, *n*^a^				
Physician specialized in pediatrics	139 (65)	81 (69)	58 (59)	0.316
Physician specialized in anesthesiology	62 (29)	32 (27)	30 (31)	
Physician specialized in general and cardiac critical ICU	44 (21)	19 (16)	25 (26)	
Nurse	20 (9)	18 (7)	12 (12)	
Physician specialized in Surgery	4 (2)	0 (0)	4 (4)	
Presence of internal protocol for analgesia and sedation	152 (71)	90 (77)	62 (63)	0.028
Use of a nurse driven protocol for analgesia and sedation (*n* = 152)	57 (38)	29 (25)	28 (29)	0.531
Nurse role in the protocol (*n* = 57 | 29 | 28)				
Choice of the drug dosage	48 (84)	24 (83)	24 (86)	0.785
Choice of the time of drug weaning	45 (79)	23 (79)	22 (79)	
Choice of the mode of drug weaning	26 (46)	14 (48)	12 (43)	
Choice of the type of drug for analgesia and sedation	22 (39)	14 (48)	8 (29)	

Data are reported as numbers and (percentages) or median and interquartile ranges according to their distribution

*ICU* intensive care unit, *PICU* pediatric ICU

^a^The sum of percentages is more than 100% because responders could indicate more than one option

### PICU characteristics and protocols

Characteristics of the PICUs are reported in Table 1. Most of the responding units were pediatric ICUs (158/215, 74%), while a minority were mixed adult and pediatric ICUs (9/215, 4%). ICUs admitting pediatric patients only were predominantly high-volume PICUs (83% vs 66%, *p* = 0.003). About one-third of the PICUs (69, 32%) admitted early post-cardiac surgery patients with a predominance in the high-volume PICUs (43% vs 23%, *p* = 0.002). Palliative care and/or terminal sedation was provided in 182 (85%) PICUs. The median number of bed capacity of the participating PICUs was 9 beds (IQR 7–16), including the smallest PICU with a capacity of two beds and the largest with 35. The median number of admissions per year was 450 (IQR 260–700). Most PICUs had an internal protocol for analgesia and sedation management (152, 71%). A protocol was more frequently used among the low-volume PICUs compared to the high-volume ones (77% vs 63%, *p* = 0.028), and was nurse-driven in 38% of cases, with the nurse being mainly responsible for adjusting the drug dosage (84%) and the timing of drug weaning (79%).

### Drug choice and dosing for analgesia and sedation

Drugs choice and dosing (starting and maximum dose) are reported in Tables 2 and 3. The drug most commonly used as first choice was fentanyl (51%) and midazolam (71%) for analgesia and sedation, respectively. Midazolam was chosen as a first-line therapy significantly more frequently in low-volume PICUs, compared to high-volume ones (77 vs 65%, *p* = 0.042). Alpha-2 agonists were only used in 18% of the PICUs as a first line agent, with a preference for dexmedetomidine over clonidine. Ketamine was more often used in high-volume PICUs compared to low-volume (16% vs 2%, *p* = 0.000). The most commonly used sedation regimen was a combination of opioids and benzodiazepines, with fentanyl and midazolam being the most common combination (38%), especially among low-volume PICUs (46% vs 28%, *p* = 0.004). Morphine and midazolam were the second most preferred combination of drugs (17%).

**Table 2:** Analgesia, sedation and paralysis drug of choice comparing PICUs according to yearly admission volume

Characteristics	Total responders *n* = 215	PICUs < 450 admissions/year *n* = 117	PICUs ≥ 450 admissions/year *n* = 98	*p* value
*Drug used as first choice for continuous analgosedation*^a^				
Opioids				
a. Fentanyl	110 (51)	64 (55)	46 (47)	0.257
b. Morphine	62 (29)	32 (27)	30 (31)	0.599
c. Sufentanil	32 (15)	16 (14)	16 (16)	0.586
d. Remifentanil	3 (1)	2 (2)	1 (1)	1.000
e. Oxycodon	1 (1)	1 (1)	0 (0)	1.000
Benzodiazepines				
a. Midazolam	153 (71)	90 (77)	63 (65)	0.042
b. Lorazepam	–	–	–	–
Alpha 2 agonists				
a. Dexmedetomidine	24 (11)	12 (10)	12 (12)	0.645
b. Clonidine	14 (7)	8 (7)	6 (6)	0.832
Others				
a. Ketamine	18 (8)	2 (2)	16 (16)	0.000
b. Propofol	5 (2)	3 (3)	2 (2)	1.000
c. Thiopentone	2 (1)	1 (1)	1 (1)	1.000
d. Chloral Hydrate	1 (1)	1 (1)	0 (0)	1.000
*Drugs used in combination as first choice*				
Fentanyl and midazolam	81 (38)	54 (46)	27 (28)	0.004
Morphine and midazolam	36 (17)	20 (17)	16 (16)	0.881
Sufentanil and midazolam	24 (11)	10 (9)	14 (14)	0.199
Fentanyl and ketamine	12 (6)	0 (0)	12 (12)	0.000
Morphine and dexmedetomidine	9 (4)	3 (3)	6 (6)	0.306
Morphine and clonidine	9 (4)	5 (4)	4 (4)	1.000
*Drug used as second choice for continuous analgosedation*^a^				
Opioids				
a. Fentanyl	57 (27)	20 (17)	37 (38)	0.001
b. Morphine	57 (27)	37 (32)	20 (20)	0.064
c. Sufentanil	12 (5)	6 (5)	6 (6)	0.774
d. Remifentanil	3 (1)	2 (2)	1 (1)	1.000
e. Methadone	2 (1)	1 (1)	1 (1)	1.000
f. Alfentanil	1 (1)	1 (1)	0 (0)	1.000
Benzodiazepines				
a. Midazolam	32 (15)	19 (16)	13 (13)	0.542
b. Lorazepam	13 (6)	7 (6)	6 (6)	0.966
Alpha 2 agonists				
a. Dexmedetomidine	117 (54)	65 (56)	52 (53)	0.715
b. Clonidine	69 (32)	31 (27)	38 (39)	0.055
Others				
a. Ketamine	120 (56)	62 (53)	58 (59)	0.363
b. Propofol	77 (36)	44 (38)	33 (34)	0.549
c. Antihistamines	13 (6)	5 (4)	8 (8)	0.262
d. Phenobarbital	4 (2)	3 (3)	1 (1)	0.627
e. Chloral Hydrate	5 (2)	2 (2)	3 (3)	0.662
f. Inhaled agents	2 (1)	2 (2)	0 (0)	0.502
*Drug used during difficult analgosedation*^a^				
Opioids				
a. Sufentanil	1 (1)	0 (0)	1 (1)	0.456
b. Remifentanil	3 (1)	2 (2)	1 (1)	1.000
c. Methadone	2 (1)	0 (0)	2 (2)	0.207
Benzodiazepines				
a. Midazolam	2 (1)	0 (0)	2 (2)	0.182
b. Lorazepam	2 (1)	1 (1)	1 (1)	1.000
Alpha 2 agonists				
a. Dexmedetomidine	74 (34)	41 (35)	33 (34)	0.833
b. Clonidine	39 (18)	19 (16)	20 (20)	0.430
Others				
a. Ketamine	109 (51)	55 (47)	54 (55)	0.237
b. Propofol	92 (43)	50 (43)	42 (43)	0.986
c. Antipsychotic agents	44 (21)	16 (14)	28 (29)	0.007
d. Chloral hydrate	35 (16)	21 (18)	14 (14)	0.469
e. Inhaled agents	34 (16)	8 (7)	26 (27)	0.000
f. Antihistamines	18 (8)	7 (6)	11 (11)	0.167
g. Thiopentone	4 (2)	3 (3)	1 (1)	0.627
h. Phenobarbital	3 (1)	2 (2)	1 (1)	1.000
*Drug never used for continuous analgosedation*^a^				
Opioids				
a. Sufentanil	105 (49)	64 (55)	41 (42)	0.060
b. Fentanyl	36 (17)	9 (8)	27 (28)	0.000
c. Morphine	15 (7)	9 (8)	6 (6)	0.653
Benzodiazepines				
a. Lorazepam	120 (56)	72 (62)	48 (49)	0.065
b. Midazolam	5 (2)	4 (3)	1 (1)	0.379
Alpha 2 agonists				
a. Clonidine	57 (27)	35 (29)	22 (22)	0.217
b. Dexmedetomidine	23 (11)	13 (11)	10 (10)	0.830
Others				
a. Inhaled agents	152 (71)	97 (83)	55 (56)	0.000
b. Antihistamines	101 (47)	62 (53)	39 (40)	0.054
c. Antipsychotic agents	90 (42)	60 (51)	30 (31)	0.002
d. Propofol	41 (19)	25 (21)	16 (16)	0.349
e. Ketamine	26 (12)	9 (8)	17 (17)	0.031
f. Chloral Hydrate	1 (1)	0 (0)	1 (1)	0.456
g. Phenobarbital	1 (1)	0 (0)	1 (1)	0.456
Use of NMBAs during analgosedation for subgroup of patients	184 (86)	96 (82)	88 (90)	0.107
*Type of NMBA used*^a^				
Rocuronium	123 (57)	73 (62)	50 (51)	0.093
Cisatracurium	58 (27)	37 (32)	21 (21)	0.093
Vecuronium	29 (14)	16 (14)	13 (13)	0.930
Succinylcholine	14 (7)	0 (0)	14 (14)	0.000
Mivacurium	5 (2)	1 (1)	4 (4)	0.180
Pancuronium	3 (1)	2 (2)	1 (1)	1.000
Pipercuronium	3 (1)	2 (2)	1 (1)	1.000
Use of paracetamol for opioid sparing	177 (82)	95 (81)	82 (84)	0.635

Data are reported as numbers and (percentages)

^a^The sum of percentages is more than 100% because responders could indicate more than one option

**Table 3:** Dosages of the drugs used as a continuous infusion for analgesia and sedation with comparison of PICUs according to yearly admission volume

Characteristics	Survey responders *n* = 215	PICUs < 450 admissions/year *n* = 117	PICUs ≥ 450 admissions/year *n* = 98	*p* value
Fentanyl, mcg/kg/h	*n* = 179	*n* = 102	*n* = 77	
Starting dose, min–max	1 (1–2), 0.1–5	1 (1–1), 0.5–5	1 (1–2), 0.1–5	0.004
Maximum dose, min–max	5 (4–7.5), 0.5–20	5 (3–5), 0.5–15	6 (5–10), 2–20	0.000
Morphine, mcg/kg/h	*n* = 154	*n* = 91	*n* = 63	
Starting dose, min–max	10 (10–20), 2–70	10 (10–20), 2–50	20 (10–30), 5–70	0.005
Maximum dose, min–max	50 (40–100), 10–500	40 (30–85), 10–500	60 (40–100), 20–500	0.025
Sufentanil, mcg/kg/h	*n* = 56	*n* = 29	*n* = 27	
Starting dose, min–max	0.2 (0.1–0.5), 0.05–3	0.2 (0.1–0.5), 0.05–1	0.3 (0.2–0.5), 0.05–3	0.106
Maximum dose, min–max	1 (0.5–2), 0.1–20	1 (0.5–2), 0.1–3	1 (0.6–2), 0.4–20	0.567
Midazolam, mg/kg/h	*n* = 192	*n* = 113	*n* = 79	
Starting dose, min–max	0.1 (0.05–0.1), 0.01–0.5	0.1 (0.06–0.1), 0.02–0.5	0.1 (0.05–0.1), 0.01–0.4	0.574
Maximum dose, min–max	0.3 (0.25–0.5), 0.05–4	6 (0.3–0.5), 0.05–2	0.3 (0.24–0.5), 0.1–4	0.436
Ketamine, mcg/kg/min	*n* = 179	*n* = 101	*n* = 78	
Starting dose, min–max	10 (5–17), 0.30–33	10 (5–17), 0.30–33	10 (5–17), 0.80–33	0.277
Maximum dose, min–max	33.3 (25–50), 3–100	33 (25–50), 3–100	33 (25–50), 3–100	0.723
Propofol, mg/kg/h	*n* = 158	*n* = 90	*n* = 68	
Starting dose, min–max	1 (1–2), 0.05–6	1 (1–2), 0.05–6	1 (1–2), 0.1–4.8	0.134
Maximum dose, min–max	4 (4–5), 0.3–20	4 (4–5), 0.4–20	4 (3–5), 0.3–15	0.271
Dexmedetomidine, mcg/kg/h	*n* = 157	*n* = 94	*n* = 63	
Starting dose, min–max	0.3 (0.2–0.5), 0.05–1	0.3 (0.2–5), 0.05–1	0.4 (0.2–0.5), 0.1–1	0.695
Maximum dose, min–max	1.2 (0.8–1.5), 0.3–5	1.2 (0.75–1.4), 0.5–4	1.4 (1–1.5), 0.3–5	0.035
Clonidine, mcg/kg/h	*n* = 99	*n* = 55	*n* = 44	
Starting dose, min–max	0.5 (0.3–0.6), 0.05–2	0.5 (0.3–0.5), 0.05–2	0.5 (0.5–1), 0.1–2	0.073
Maximum dose, min–max	2 (2–2.4), 0.3–10	2 (2–2.75), 0.3–10	2 (2–2), 0.5–8	0.759

Data are reported as median and interquartile ranges according to their distribution

Among the responding PICUs, the drugs most commonly used as a second choice for analgesia and sedation were ketamine (56%), and dexmedetomidine (54%). In difficult-to-sedate cases, the top three drugs used were ketamine (51%), propofol (43%) and dexmedetomidine (34%). In high-volume PICUs, antipsychotic and inhaled agents were more often used than in low-volume PICUs (*p* = 0.007 and *p* < 0.001, respectively). Inhaled agents were reported never to be used for continuous analgesia and sedation by 71% of the responders. Furthermore, 49% of the PICUs did not administer sufentanil among opioids, 56% did not use lorazepam among benzodiazepines and 27% did not prescribe clonidine among alpha-2 agonists. More responders from high-volume PICUs reported they never used fentanyl (28% vs 8% *p* < 0.001), while responders from low-volume PICUs more often stated their unit never use inhaled agents (83% vs 56%, 0.000) and antipsychotic agents (51% vs 31%, *p* = 0.002). Paracetamol was reported to be used as an opioid sparing drug in 177 (82%) PICUs.

The vast majority of the responders (86%) stated that they used NMBAs during analgesia and sedation for a subgroup of patients, with rocuronium (57%) being the preferred paralysing agent used. Succinylcholine was more frequently used in high-volume PICUs (14% vs 0%, *p* = 0.000).

Table 3 reports the median doses with their respective interquartile ranges as well as the minimum and maximum doses of the drugs used as continuous infusion for analgesia and sedation in the PICUs. High volume PICUs demonstrated a higher starting dose of fentanyl (1 mcg/kg/h [IQR 1–2] vs  mcg/kg/h [IQR 1–1], *p* = 0.004 and morphine (20 mcg/kg/h [IQR 10–30] vs 10 mcg/kg/h [IQR 10–20], *p* = 0.005) as well as a higher maximum dose of fentanyl (6 mcg/kg/h [IQR 5–10] vs 5 mcg/kg/h [IQR 3–5], *p* < 0.001), morphine (60 mcg/kg/h [IQR 40–100] vs 40 mcg/kg/h [IQR 30–85]) and dexmedetomidine (1.4 mcg/kg/h [IQR 1–1.5] vs 1.2 mcg/kg/h [IQR 0.8–1.5], *p* = 0.035). Of note, minimum and maximum starting and maximum doses ranges varied widely, such as from 0.1 to 5 mcg/kg/h for fentanyl and 0.01 to 0.5 mg/kg/h for midazolam.

### Pain and sedation assessment and monitoring

**Fig. 2:**
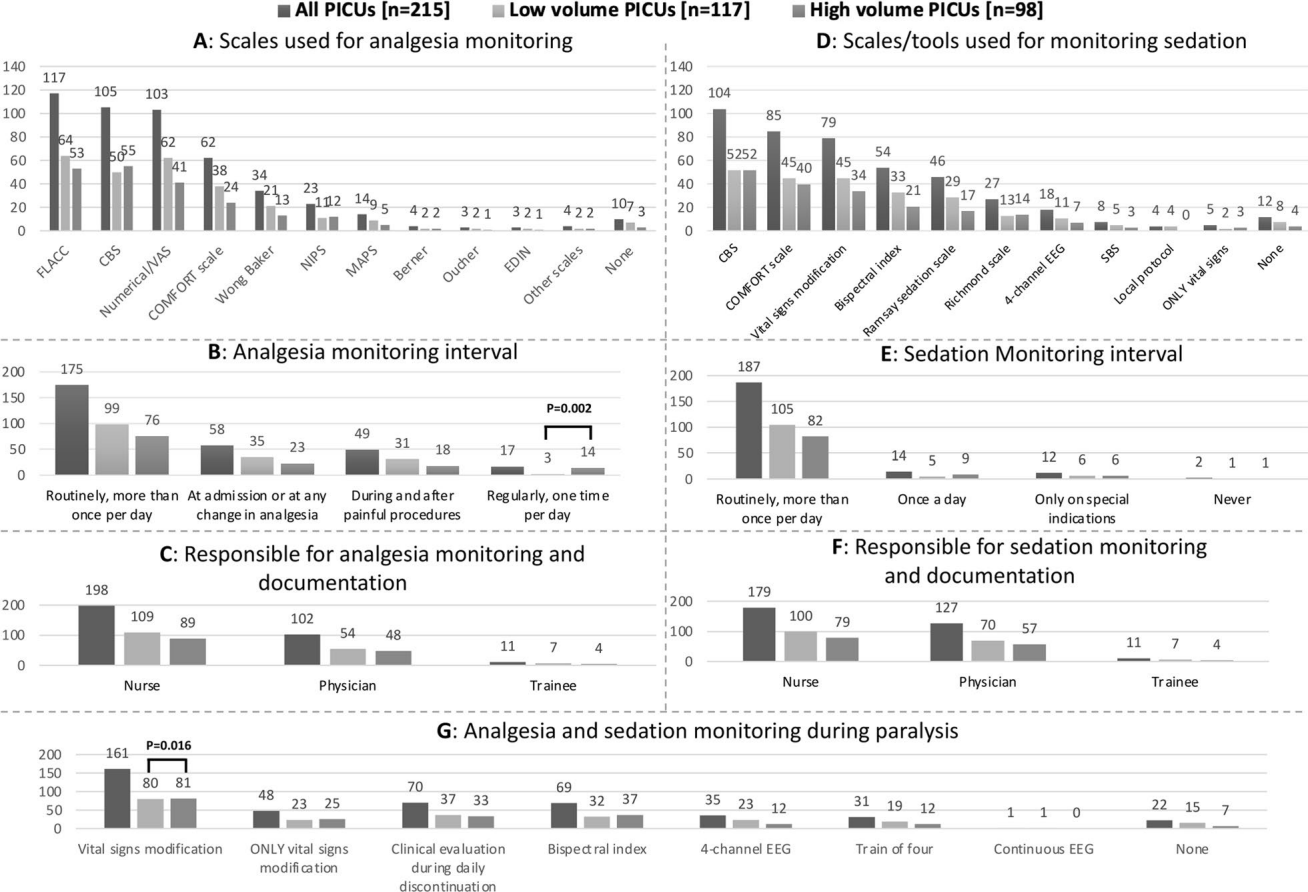
**A–G** Analgesia and sedation assessment and monitoring with comparison of PICUs according to their yearly admission volume. *CBS* COMFORT Behavioral Scale, *EDIN* Échelle Douleur Inconfort Nouveau-Né, *FLACC* Faces, Legs, Activity, Cry and Consolability, *MAPS* Multidimensional Assessment Pain Scale, *NIPS* Neonatal Infant Pain Scale, *SBS* State Behavioral Scale, *VAS* Visual Analogic Scale

Pain and sedation assessment and monitoring are reported in Fig. 2. Pain was assessed with three different scales mainly: Faces, Legs, Activity, Cry and Consolability (FLACC) scale was reported by 117 responders (54%), COMFORT Behavioral (COMFORT-B) scale by 105 (49%), and numerical/visual analogue scale by 103 (48%); ten PICUs (5%) reported they did not monitor pain and analgesia at all (Fig. 2A). Responders reported that pain was monitored and documented routinely more than one time per day in 175 PICUs (81%) (Fig. 2B).

Sedation was routinely monitored mainly with COMFORT-B scale (104, 48%), COMFORT Scale (85, 40%) and vital signs modification (79, 37%) and documented more than one time per day in 187 (87%) of the PICUs (Fig. 2D, E). Five PICUs (2%) used only monitoring of vital signs alteration as sedation monitoring. Twelve PICUs (6%) reported they did not monitor the level of sedation at all.

Both analgesia and sedation were mainly monitored by nurses (92% and 84%, respectively) compared to physicians (47% and 60% respectively) (Fig. 2C, F). For analgesia, monitoring was performed exclusively by nurses in 113 PICUs (53%) and by physicians in 17 PICUs (8%). For sedation, monitoring was performed exclusively by nurses in 85 PICUs (40%) and by physicians in 34 PICUs (16%).

Patients’ analgesia and sedation monitoring during paralysis was mostly a combination of assessments and mainly based on vital signs modification (161, 75%), while clinical evaluation during daily discontinuation was used in 33% of the PICUs. The monitoring using electronic devices was used in a minority of the responding units and were: bispectral index (69, 32%), 4-channel processed EEG (35, 16%), and peripheral nerve train-of-four (31, 14%). Forty-eight PICUs (22%) used the observation of vital signs modification only, while 22 PICUs (10%) reported they did not monitor the level of analgesia and sedation during paralysis.

## Discussion

This cross-sectional survey reports the current state of analgesia and sedation management among European PICUs with a comparison between high- and low-volume units. The preferred first-choice opioid was found to be fentanyl, followed by morphine. The preferred first-choice benzodiazepine was found to be midazolam. This is in line with a preceding survey on sedation management which included more than 300 participants mostly from the USA [14].

Previous studies reported that benzodiazepines are independent risk factors for delirium [16, 17]; therefore, the replacement of benzodiazepines in favour for alternative drugs like alpha-2 agonists has been advocated [18]. The recently published clinical practice guidelines for the management of Pain, Agitation, Neuromuscular Blockade, and Delirium in critically ill pediatric patients with consideration of the PICU Environment and Early Mobility (PANDEM) and the Recommendations for analgesia and sedation in critically ill children admitted to intensive care unit [8, 19] both recommend the use of alpha_2_-agonists over benzodiazepines as a first-line sedative. It has been shown that alpha_2_-agonists is a safe and benzodiazepine sparing alternative for sedation in the pediatric population [20–22]. We could reveal that only as much as 11% of the responding PICUs in Europe would use dexmedetomidine as a first-choice sedative and only 7% opted for clonidine. However, as a second-choice sedative dexmedetomidine was reported to be prescribed by more than half of the respondents.

Propofol, being known to possibly cause severe side effects in children [6], did not play a role in first-choice sedation, but its usage was reported both as second choice (36%) or in difficult sedation scenarios (43%) by the responding PICUs. The same tendency could be found for ketamine, antipsychotics and inhaled agents. Access to inhaled agents as an option in difficult sedation scenarios seems to be more feasible for high-volume PICUs. Even though inhaled agents have been found to be an effective alternative to intravenous sedation [23], concerns about toxicity and long-term neurologic impairment have been voiced [24, 25]. Given the current lack of evidence for safe long-term usage in PICU patients, inhaled agents should remain to be an option in difficult sedation scenarios in absence of probably less toxic alternatives.

Concerning drug dosing, we found median starting doses to be relatively low in comparison with previously published dosing recommendations [6]. The obtained data from our survey can serve as an orientation for further dosing recommendations. It has to be mentioned that among all respondents starting and maximum doses differed widely. A study comparing opioid doses in children all undergoing stage 1 palliation for hypoplastic left heart syndrome in five North American cardiac PICUs showed more than fourfold differing median opioid doses between the centres [26]. Even though sedation requirements may differ between different PICUs and their specialties, these wide ranges imply the need for adequate dosing recommendations to avoid under- and over-sedation in pediatric patients as well as potential related side effects [27, 28]. We found that starting and maximum doses of both fentanyl and morphine were significantly higher in larger PICUs. Although it is not possible to draw any conclusion from our data, high-volume PICUs can be expected to admit populations with more clinically complex needs, requiring deeper or longer analgosedation with a higher probability of developing tolerance.

The ESPNIC position statement on recommendations for pain, sedation, withdrawal and delirium assessment in critically ill infants and children provided several recommendations on assessing pain and sedation with the ultimate goal to achieve “the best possible treatment for pain, distress, inadequate sedation, withdrawal syndrome and delirium” [10]. In our survey, about three out of four PICUs responded to have a sedation and analgesia guideline in place and only 5% stated they would not use any assessment tool at all. To evaluate analgesia in children and to achieve a “common language” it has been recommended to assess pain with an age-appropriate and validated instrument [11]. The most common tools for analgesia assessments in responding European PICUs were the FLACC scale, the COMFORT-B scale, the COMFORT scale and visual analogue scales (VAS), all being validated instruments. As much as 81% of the PICUs assess pain and analgesia more than once per day; yet only 27% of them stated to undertake assessments before and after changes in their patients’ analgesia. This implies that although pain assessments are undertaken regularly, they are not performed to titrate analgesic medication systematically by most of the responding PICUs leading to the risk of over- or underdosing. To verify treatment effects and drive further measurements it has been recommended to reassess frequently after interventions [18]. The COMFORT B scale for example is feasible to detect changes after pain-treatment [29].

Use of validated instruments has also been recommended to achieve optimal sedation levels [30–32]. The most common tool used among European PICUs was found to be the COMFORT-B and the COMFORT scale. Important to underline, only a minimal percentage of the responding PICUs did not monitor sedation at all or used only vital signs for its assessment. We strongly encourage an effort in education in using appropriate validated tools for sedation levels.

While nearly all PICUs prescribe NMBAs, assessment of sedation level during paralysis seems to be achieved with variable methods. Most of the responding PICUs reported to monitor paralysis by vital signs, some stated to use specific devices such as bispectral index and/or processed EEG. Without standardized monitoring, paralysis with its possible detrimental side effects [33, 34] is left to the perception and knowledge of the individual caretaker and is prone to inconsistency. This again emphasizes the need of clear guidelines on this topic.

A major part of the survey responders stated to have an internal protocol for driving sedation and about one-third of the PICUs would use a nurse driven sedation protocol. Over the last 2 decades use of nurse-driven sedation protocols has been increasingly reported and evaluated [35–38]. A recently published study showed a significant reduction of time to successful extubation after implementation of a sedation and ventilator liberation protocol in 18 PICU sites [39]. However, its clinical importance may be questionable since the median time to extubation would only decrease from 66.2 to 64.8 h after the implementation of the protocol intervention. A previous large cluster RCT conducted in 31 PICUs could not show a significant reduction of ventilator time after the implementation of nurse goal-directed sedation protocol, but intubated patients spent significantly more days awake and calm while being intubated [40]. Other studies could show a reduction of opioids and benzodiazepines [41, 42], as well as a reduction in PICU length of stay with implementation of nurse-driven protocolized sedation [43]. Whether the use of nurse-driven sedation protocols should be implemented or not, could be debated, but the abovementioned evidence show more advantages than drawbacks with such an approach.

This survey still leaves some open questions to be answered. We did not assess if analgosedation is always done by continuous infusions or if some PICUs prefer intermittent boluses. Furthermore, we did not include questions about daily sedation interruption, a practice whose benefit is still to be determined in the PICU patient [44]. We did not specify the type of analgosedation protocols and did not assess for the units’ compliance towards those protocols and validated assessment tools. We did not obtain data on dosing of NMBAs. In order to achieve a safe approach towards paralysis we advocate for further research and development of dosing and monitoring guidelines.

Some limitations must be addressed. First, we could not collect data from all European countries, especially from the Eastern part of Europe. This was partly due to the lack of contacts and knowledge about the respective infrastructure, language barriers, and missing responses. Nevertheless, we had a response rate of 60%, which is notable; thus, we believe this survey provides a good overview on common sedation practices across Europe. Second, differing ranges in drug dosing might be accorded to different patient populations; we did not only include exclusive pediatric ICUs and we did not retrieve data for respective specialties. Third, in order to avoid duplicates in the responses, we contacted one person (physician, nurse) only in each PICU. Nevertheless, the answers of the respondents might not perfectly reflect common practice in her/his unit. Fourth, although the survey was validated for clarity, some wordings might have been open for different interpretations, such as “difficult sedation” or “prolonged continuous sedation”. Fifth, we decided to divide all the units included in high- and low- volume PICUs according to a cut-off of 450 admissions per year possibly leading to a selection bias. Without any objective a priori definition of these two groups, we tried to use the median number of admissions in our population as a criterion being as objective as possible. We do, however, acknowledge that this may have led to a selection bias possibly limiting the external validity of our results. Last, the possibility of responder bias could not be avoided, being inherent to questionnaires of this kind.

## Conclusion

This survey provides an overview of current analgosedation practices among European PICUs in 27 countries. For the first time we were able to document common starting and maximum doses of the most commonly used analgesic and sedative drugs. Dosing and assessment strategies differ widely between European PICUs and between low- and high-volume PICUs. Further research on evidence-based guidelines for optimal drug dosing and analgosedation assessment in the individual patient through primary and secondary endpoints is necessary to enhance the level of current evidence-based guidelines and ultimately improve the respective patient’s comfort and outcome.

## Supplementary Information

**Additional file 1**. Survey questions.

## Data Availability

The datasets used and/or analysed during the current study are available from the corresponding author on reasonable request.

## References

[1] Aranda JV (2020). Neonatal and pediatric pharmacology: therapeutic principles in practice.

[2] Egbuta C, Mason KP (2021). Current state of analgesia and sedation in the pediatric intensive care unit. J Clin Med.

[3] Ista E, Van Dijk M, Gamel C, Tibboel D, De Hoog M (2008). Withdrawal symptoms in critically ill children after long-term administration of sedatives and/or analgesics: a first evaluation. Crit Care Med.

[4] Choong K (2019). Picu-acquired complications: the new marker of the quality of care. ICU Manag Pract.

[5] Association of Paediatric Anaesthetists of Great Britain and Ireland. Good Practice in Postoperative and Procedural Pain Management 2nd Edition. Pediatr Anesth. 2012. 10.1111/j.1460-9592.2012.3838.x. 10.1111/j.1460-9592.2012.03838.x 22817132

[6] Playfor S, Jenkins I, Boyles C (2006). Consensus guidelines on sedation and analgesia in critically ill children. Intensive Care Med.

[7] Playfor S, Jenkins I, Boyles C (2007). Consensus guidelines for sustained neuromuscular blockade in critically ill children. Paediatr Anaesth.

[8] Smith HAB, Besunder JB, Betters KA (2022). 2022 society of critical care medicine clinical practice guidelines on prevention and management of pain, agitation, neuromuscular blockade, and delirium in critically ill pediatric patients with consideration of the ICU environment and early mobility. Pediatr Crit Care Med.

[9] Royal Collage of Nursing. The recognition and assessment of acute pain in children; 2009.

[10] Harris J, Ramelet AS, van Dijk M (2016). Clinical recommendations for pain, sedation, withdrawal and delirium assessment in critically ill infants and children: an ESPNIC position statement for healthcare professionals. Intensive Care Med.

[11] Giordano V, Edobor J, Deindl P (2019). Pain and sedation scales for neonatal and pediatric patients in a preverbal stage of development: a systematic review. JAMA Pediatr.

[12] Koizumi T, Kurosawa H (2020). Survey of analgesia and sedation in pediatric intensive care units in Japan. Pediatr Int.

[13] Tabacco B, Tacconi C, Amigoni A (2017). Survey on monitoring analgesia and sedation in the Italian pediatric intensive care units. Minerva Anestesiol.

[14] Kudchadkar SR, Yaster M, Punjabi NM (2014). Sedation, sleep promotion, and delirium screening practices in the care of mechanically ventilated children: a wake-up call for the pediatric critical care community. Crit Care Med.

[15] Burns KEA, Duffett M, Kho ME (2008). A guide for the design and conduct of self-administered surveys of clinicians. CMAJ.

[16] Traube C, Silver G, Reeder RW (2017). Delirium in critically ill children: an international point prevalence study. Crit Care Med.

[17] Mody K, Kaur S, Mauer EA (2018). Benzodiazepines and development of delirium in critically ill children: estimating the causal effect. Crit Care Med.

[18] Walz A, Canter MO, Betters K (2020). The ICU liberation bundle and strategies for implementation in pediatrics. Curr Pediatr Rep.

[19] Amigoni A, Conti G, Conio A (2022). Recommendations for analgesia and sedation in critically ill children admitted to intensive care unit. J Anesth Analg Crit Care.

[20] Sperotto F, Mondardini MC, Dell’Oste C (2020). Efficacy and safety of dexmedetomidine for prolonged sedation in the PICU: a prospective multicenter study (PROSDEX). Pediatr Crit Care Med.

[21] Erickson SJ, Millar J, Anderson BJ (2020). Dexmedetomidine sedation in mechanically ventilated critically ill children: a pilot randomized controlled trial. Pediatr Crit Care Med.

[22] Daverio M, Sperotto F, Zanetto L (2020). Dexmedetomidine for prolonged sedation in the PICU: a systematic review and meta-analysis∗. Pediatr Crit Care Med.

[23] Mencía S, Palacios A, García M (2018). An exploratory study of sevoflurane as an alternative for difficult sedation in critically ill children. Pediatr Crit Care Med.

[24] de Graaff JC, Houmes RJ, Tibboel D (2018). Navigating between Scylla and Charybdis; sevoflurane for difficult sedation at the PICU. Pediatr Crit Care Med.

[25] Andropoulos DB, Greene MF (2017). Anesthesia and developing brains—implications of the FDA warning. N Engl J Med.

[26] Sperotto F, Davidson JA, Smith-Parrish MN (2021). Development of care curves following the stage 1 palliation: a comparison of intensive care among 5 centers. J Am Heart Assoc.

[27] Dreyfus L, Javouhey E, Denis A, Touzet S, Bordet F (2017). Implementation and evaluation of a paediatric nurse-driven sedation protocol in a paediatric intensive care unit. Ann Intensive Care.

[28] Vet NJ, Ista E, De Wildt SN, Van Dijk M, Tibboel D, De Hoog M (2013). Optimal sedation in pediatric intensive care patients: a systematic review. Intensive Care Med.

[29] Boerlage AA, Ista E, Duivenvoorden HJ, De Wildt SN, Tibboel D, Van Dijk M (2015). The COMFORT behaviour scale detects clinically meaningful effects of analgesic and sedative treatment. Eur J Pain (United Kingdom).

[30] Keogh SJ, Long DA, Horn DV (2015). Practice guidelines for sedation and analgesia management of critically ill children: a pilot study evaluating guideline impact and feasibility in the PICU. BMJ Open.

[31] Ista E, Van Dijk M, Tibboel D, De Hoog M (2005). Assessment of sedation levels in pediatric intensive care patients can be improved by using the COMFORT “behavior” scale. Pediatr Crit Care Med.

[32] Westcott C (1995). The sedation of patients in intensive care units: a nursing review. Intensive Crit Care Nurs.

[33] Glau CL, Conlon TW, Himebauch AS (2018). Progressive diaphragm atrophy in pediatric acute respiratory failure*. Pediatr Crit Care Med.

[34] Guess R, Vaewpanich J, Coss-Bu JA (2018). Risk factors for ventilator-associated events in a PICU*. Pediatr Crit Care Med.

[35] Deeter KH, King MA, Ridling D, Irby GL, Lynn AM, Zimmerman JJ (2011). Successful implementation of a pediatric sedation protocol for mechanically ventilated patients. Crit Care Med.

[36] Ista E, De Hoog M, Tibboel D, Van Dijk M (2009). Implementation of standard sedation management in paediatric intensive care: effective and feasible?. J Clin Nurs.

[37] Gaillard-Le Roux B, Liet JM, Bourgoin P, Legrand A, Roze JC, Joram N (2017). Implementation of a nurse-driven sedation protocol in a PICU decreases daily doses of midazolam. Pediatr Crit Care Med.

[38] Neunhoeffer F, Kumpf M, Renk H (2015). Nurse-driven pediatric analgesia and sedation protocol reduces withdrawal symptoms in critically ill medical pediatric patients. Paediatr Anaesth.

[39] Blackwood B, Tume LN, Morris KP (2021). Effect of a sedation and ventilator liberation protocol vs usual care on duration of invasive mechanical ventilation in pediatric intensive care units. JAMA.

[40] Curley MAQ, Wypij D, Watson RS (2015). Protocolized sedation vs usual care in pediatric patients mechanically ventilated for acute respiratory failure: a randomized clinical trial. JAMA J Am Med Assoc.

[41] Lincoln PA, Whelan K, Hartwell LP (2020). Nurse-implemented goal-directed strategy to improve pain and sedation management in a pediatric cardiac ICU. Pediatr Crit Care Med.

[42] Michel J, Hofbeck M, Peper AK, Kumpf M, Neunhoeffer F (2020). Evaluation of an updated sedation protocol to reduce benzodiazepines in a pediatric intensive care unit. Curr Med Res Opin.

[43] Hanser A, Neunhoeffer F, Hayer T (2020). A nurse-driven analgesia and sedation protocol reduces length of PICU stay and cumulative dose of benzodiazepines after corrective surgery for tetralogy of Fallot. J Spec Pediatr Nurs.

[44] Vet NJ, de Wildt SN, Verlaat CWM (2016). A randomized controlled trial of daily sedation interruption in critically ill children. Intensive Care Med.

