# Correction to: Pain and sedation management and monitoring in pediatric intensive care units across Europe: an ESPNIC survey

**DOI:** 10.1186/s13054-022-03992-4

**Published:** 2022-05-16

**Authors:** Marco Daverio, Florian von Borell, Anne-Sylvie Ramelet, Francesca Sperotto, Paula Pokorna, Sebastian Brenner, Maria Cristina Mondardini, Dick Tibboel, Angela Amigoni, Erwin Ista, the Analgosedation CONSORTIUM on behalf of the Pharmacology Section and the Nurse Science Section of the European Society of Paediatric and Neonatal Intensive Care, Ermira Kola, Maria Vittinghoff, Elim Duval, Branka Polić, Frédéric Valla, Felix Neunhoeffer, Tziouvas Konstantinos, Zoltán Györgyi, Mong Hoi Tan, Antigona Hasani, Edita Poluzioroviene, Reinis Balmaks, Mickael Afanetti, Gunnar Bentsen, Alicja Bartkowska-Sniatkowska, Cristina Camilo, Dusica Simic, Yolanda M. López-Fernández, Janet Mattsson, Hasan Özen, Dmytro Dmytriiev, Joseph C. Manning, Hakan Tekgüç

**Affiliations:** 1 Pediatric Intensive Care Unit, Department of Woman’s and Child’s Health, University Hospital of Padua, Padua, Italy; 2 Department of Pediatric Cardiology and Intensive Care Medicine, Hannover Medical School, Hannover, Germany; 3 Institute of Higher Education and Research in Healthcare, Faculty of Biology and Medicine, University of Lausanne, Lausanne, Switzerland; 4 Department Woman-Mother-Child, Lausanne University Hospital, Lausanne, Switzerland; 5 Department of Cardiology, Boston Children’s Hospital, Harvard Medical School, Boston, MA USA; 6 Institute of Pharmacology, First Faculty of Medicine, Charles University and General University Hospital, Prague, Czech Republic; 7 Department of Paediatrics and Inherited Metabolic Disorders, First Faculty of Medicine, Charles University and General University Hospital, Prague, Czech Republic; 8 Intensive Care and Department of Paediatric Surgery, Erasmus Medical Center Sophia Children’s Hospital, Rotterdam, The Netherlands; 9 Department of Physiology and Pharmacology, Karolinska Institutet and Karolinska University Hospital, Stockholm, Sweden; 10 Department of Pediatric and Adolescent Medicine, University Hospital Dresden, Dresden, Germany; 11 Pediatric Anesthesia and Intensive Care Unit, Department of Woman’s and Child’s Health, IRCCS University Hospital of Bologna Policlinico S.Orsola, Bologna, Italy; 12 Department of Woman’s and Child’s Health, IRCCS University Hospital of Bologna Policlinico S.Orsola, Bologna, Italy; 13 Pediatric Intensive Care Unit, Department of Pediatric Surgery, Erasmus MC – Sophia Children’s Hospital, University Medical Center Rotterdam, Rotterdam, The Netherlands; 14 University Hospital Centre, Tirana, Albania; 15 Department of Anaesthesiology and Intensive Care Medicine, Medical University, Graz, Austria; 16 Antwerp University Hospital, Antwerp Edegem, Belgium; 17 Paediatric Intensive Care Unit Department of Paediatrics, University Hospital of Split, Split, Croatia; 18 Paediatric Intensive Care Hôpital Femme Mère Enfant, Hospices Civils de Lyon, Lyon, France; 19 Department of Pediatric Cardiology, Pulmonology and Intensive Care Medicine, University Children’s Hospital, Tübingen, Germany; 20 Paediatric Intensive Care Unit Aglaia and Panagiotis, Kyriakou Children’s Hospital, Athens, Greece; 21 2nd Department of Pediatrics, Semmelweis University, Budapest, Hungary; 22 Pediatric Intensive Care Unit, Children’s Health Ireland, Crumlin, Ireland; 23 Department of Anesthesiology, Reanimation and Emergency Medicine Faculty of Medicine, University of Prishtina, Prishtina, Kosovo Serbia; 24 Vilnius University Hospital Santaros Klinikos, Vilnius, Lithuania; 25 Intensive Care Unit, Children’s Clinical University Hospital, Riga, Latvia; 26 Pediatric Intensive Care Unit, Hôpitaux Pédiatriques de Nice, Nice, France; 27 Department of Anaesthesiology, Oslo University Hospital, Rikshospitalet, Norway; 28 Department of Paediatric Anaesthesiology and Intensive Therapy, Poznan University of Medical Sciences, Poznan, Poland; 29 Paediatric Intensive Care Unit, Department of Paediatrics, Hospital de Santa Maria - CHULN, Lisbon, Portugal; 30 University Children’s Hospital Medical Faculty, University of Belgrade, Belgrade, Serbia; 31 Cruces University Hospital Biocruces-Bizkaia Health Research Institute, Basque Country, Spain; 32 Intensive Care Red Cross University, Stockholm, Sweden; 33 Department of Pediatric Critical Care Medicine, Ankara University Faculty of Medicine, Ankara, Turkey; 34 Department of Anesthesiology and Intensive Care, Vinnitsa National Medical University, Vinnytsia Oblast, Ukraine; 35 Nottingham Children’s Hospital, University of Nottingham, Nottingham, UK; 36 Paediatric Intensive Care Unit, Department of Pediatrics, Dr. Burhan Nalbantoglu State Hospital, Lefkosa, North Cyprus Turkey

## Correction to: Critical Care (2022) 26:88 10.1186/s13054-022-03957-7

Following publication of the original article [1], the authors identified an error in the author name Marco Daverio. The given name and family name were erroneously transposed.

The incorrect author name is: Daverio Marco.

The correct author name is: Marco Daverio.

The incorrect Results section in the Abstract reads: Seventy-one percent of PICUs stated to use protocols for analgosedation management, more frequently in high-volume PICUs (77% vs 63%, *p* = 0.028).

It should instead read: Seventy-one percent of PICUs stated to use protocols for analgosedation management, more frequently in low-volume PICUs (77% vs 63%, *p* = 0.028).

The author group has been updated above, and the original article [1] has been corrected.
