# Supplementary material for: Pain and sedation management and monitoring in pediatric intensive care units across Europe: an ESPNIC survey
Source: Crit Care. 2022 Mar 31;26:88. doi: 10.1186/s13054-022-03957-7 (PMC8969245; doi:10.1186/s13054-022-03957-7)
Supplement: Supplementary file 1 — Additional file 1. Survey questions. [file 13054_2022_3957_MOESM1_ESM.docx]

**Appendix A:**

**Survey of Analgesia and Sedation Practice in European Pediatric Intensive Care Units**

**Part A: PICU and Patients characteristics**

1. **Institution Country**
2. **Institution City**
3. **Institution Name**
4. **What type of PICU do you work in? (Please select all the adequate)**

⬜ Pediatric ICU

⬜ Mixed Neonatal and Pediatric ICU

⬜ Mixed Adult and pediatric ICU

1. **Does your PICU admit patients immediately post-cardiac surgery (first 24-48 hours)?**

⬜ Yes

⬜ No

1. **Insert the maximum PICU bed capacity of your Institution? (insert number NOT in letters)**_____
2. **How many admissions do you have per year in your PICU (average in the last 3 years)?**
3. **Is your institution considered an academic/teaching hospital (i.e. Residents/Fellows participate in patient care?)**

⬜ Yes

⬜ No

1. **Does your PICU provide palliative care/terminal sedation for any patient?**

⬜ Yes

⬜ No

1. **What is your professional specialty? (Please select all the adequate)**

⬜ MD, specialized in General or Cardiac Critical Care

⬜ MD, specialized in Anesthesiology

⬜ MD, specialized in Pediatrics

⬜ MD, specialized in Surgery

⬜ Nurse

⬜ Nurse Practitioner

⬜ Research Nurse

**PART B. Analgesia & Sedation practice**

1. **Does your PICU have a protocol for continuous prolonged analgosedation?**

⬜ Yes

⬜ No

⬜ Not sure

1. **Among the following, which is (are) your first line drug for continuous prolonged analgosedation? (maximum two answers allowed)**

⬜ Fentanyl

⬜ Morphine

⬜ Sufentanil

⬜ Midazolam

⬜ Lorazepam

⬜ Ketamine

⬜ Propofol

⬜ Dexmedetomidine

⬜ Clonidine

⬜ Other

1. **If you selected “Other to the previous question, please specify**
2. **Among the following, which is (are) your second line drug(s) for continuous prolonged analgosedation? (Please select all the adequate)**

⬜ Fentanyl

⬜ Morphine

⬜ Sufentanil

⬜ Midazolam

⬜ Lorazepam

⬜ Ketamine

⬜ Propofol

⬜ Dexmedetomidine

⬜ Clonidine

⬜ Antihistamines

⬜ Other

1. **If you selected “Other to the previous question, please specify**
2. **Among the following, which drug (s) do you use only in difficult continuous prolonged analgosedations? (please select all the adequate)**

⬜ Propofol

⬜ Dexmedetomidine

⬜ Clonidine

⬜ Antipsychotic agents

⬜ Inhaled sevoflurane

⬜ Chloralum Hydratum

⬜ Ketamine

⬜ Antihistamines

⬜ Other

1. **If you selected “Other to the previous question, please specify**
2. **Among the following, is there any drug you never use in your Unit for continuous prolonged analgosedation? (Please select all the adequate)**

⬜ Fentanyl

⬜ Morphine

⬜ Sufentanil

⬜ Midazolam

⬜ Lorazepam

⬜ Ketamine

⬜ Propofol

⬜ Dexmedetomidine

⬜ Clonidine

⬜ Inhaled Sevoflurane

⬜ Antipsychotic agents

⬜ Antihistamines

⬜ Other

1. **If you selected “Other to the previous question, please specify**
2. **In your PICU is paracetamol used for opioid sparing?**

⬜ Yes

⬜ No

⬜ Not sure

1. **In your unit, if you use FENTANYL, what is the starting dose for continuous prolonged analgosedation in mcg/kg/h?**
2. **In your unit, if you use FENTANYL, what is the maximum dose for continuous prolonged analgosedation in mcg/kg/h?**
3. **In your unit, if you use MORPHINE, what is the starting dose for continuous prolonged analgosedation in mcg/kg/h?**
4. **In your unit, if you use MORPHINE, what is the maximum dose for continuous prolonged analgosedation in mcg/kg/h?**
5. **In your unit, if you use SUFENTANIL, what is the starting dose for continuous prolonged analgosedation in mcg/kg/h?**
6. **In your unit, if you use SUFENTANIL, what is the maximum dose for continuous prolonged analgosedation in mcg/kg/h?**
7. **In your unit, if you use MIDAZOLAM, what is the starting dose for continuous prolonged analgosedation in mg/kg/h?**
8. **In your unit, if you use MIDAZOLAM, what is the maximum dose for continuous prolonged analgosedation in mg/kg/h?**
9. **In your unit, if you use LORAZEPAM, what is the starting dose for continuous prolonged analgosedation in mg/kg/h?**
10. **In your unit, if you use LORAZEPAM, what is the maximum dose for continuous prolonged analgosedation in mg/kg/h?**
11. **In your unit, if you use KETAMINE, what is the starting dose for continuous prolonged analgosedation in mg/kg/h?**
12. **In your unit, if you use KETAMINE, what is the maximum dose for continuous prolonged analgosedation in mg/kg/h?**
13. **In your unit, if you use PROPOFOL, what is the starting dose for continuous prolonged analgosedation in mg/kg/h?**
14. **In your unit, if you use PROPOFOL, what is the maximum dose for continuous prolonged analgosedation in mg/kg/h?**
15. **In your unit, if you use DEXMEDETOMIDINE, what is the starting dose for prolonged continuous analgosedation in mcg/kg/h?**
16. **In your unit, if you use DEXMEDETOMIDINE, what is the maximum dose for prolonged continuous analgosedation in mcg/kg/h?**
17. **In your unit, if you use CLONIDINE, what is the starting dose for continuous prolonged analgosedation in mcg/kg/h?**
18. **In your unit, if you use CLONIDINE, what is the maximum dose for continuous prolonged analgosedation in mcg/kg/h?**
19. **In your Unit, do you use NEUROMUSCOLAR BLOCKING AGENTS as prolonged continuous infusion in sedated patients?**

⬜ Yes

⬜ No

1. **Among the following, which type of NEUROMUSCULAR BLOCKING AGENT (NMBA) do you use for prolonged continuous infusion?**

⬜ Rocuronium

⬜ Vecuronium

⬜ Atracurium

⬜ Cisatracurium

⬜ Mivacurium

⬜ Pancuronium

⬜ Succinilcoline

⬜ Others

⬜ We do not use NMBA

1. **If you selected “Other to the previous question, please specify**
2. **Among the following, which type of MONITORING do you use in your Unit for patients receiving NMBAs for prolonged continuous analgosedation? (Please select all the adequate)**

⬜ Vital signs modification

⬜ Peripheral nerve stimulation

⬜ Bispectral index

⬜ 4-channel processed EEG

⬜ Clinical scale evaluation during daily discontinuation of NMBA infusion

⬜ None

⬜ Other

1. **If you selected “Other” to the previous question, please specify:**
2. **For sedation drug administration, do you use a nurse driven protocol?**

⬜ Yes

⬜ No

1. **If you have a nurse driven protocol, it involves: (select all that apply)**

⬜ I confirm I don’t have a nurse driven protocol

⬜ The choice of the drug

⬜ The choice of the dosage

⬜ The choice of the time of weaning

⬜ The choice of modality of weaning

⬜ I don’t have a nurse driven protocol

**PART C. Analgesia and Sedation monitoring**

1. **In your Unit, do you use any tool/scale for PAIN/ANALGESIA monitoring? (Please select all the adequate)**

⬜ FLACC scale /

⬜ Wong-Baker scale

⬜ Oucher scale

⬜ MAPS (Multidimensional Assessment Pain Scale)

⬜ Numerical Rating Scale

⬜ COMFORT Behavior Scale

⬜ COMFORT Scale

⬜ PIPP/PIPP Revised

⬜ NIPS

⬜ None

⬜ Other

1. **If you selected “Other” to the previous question, please specify:**
2. **In your Unit, how often do you monitor PAIN/ANALGESIA? (Please select all the adequate)**

⬜ Routinely, more than one time per day

⬜ Regularly, one time per day

⬜ After new admissions or before/after any change in analgesi

⬜ Before, during, and after painful procedure

⬜ Other (please specify)___________

⬜ Never

1. **In your Unit, who is responsible for PAIN/ANALGESIA evaluation and documentation?**

⬜ Nurse

⬜ Physician

⬜ Trainee

⬜ Other

1. **If you selected Other to the previous question, please specify:**
2. **In your Unit, do you use any tool/scale/devices for SEDATION level monitoring? (Please select all the adequate)**

⬜ Comfort Scale

⬜ Comfort Behavioral Scale

⬜ State Behavior Scale

⬜ Richmond Sedation Agitation Scale

⬜ Ramsay Sedation Scale

⬜ Local/Internal scales

⬜ Bispectral index

⬜ 4-channel processed EEG

⬜ Vital signs modification

⬜ None

⬜ Other

1. **If you selected “Other” to the previous question, please specify:**
2. **In your Unit, how often do you assess level of SEDATION?**

⬜ Regularly, more than one time per day

⬜ Regularly, one time per day

⬜ Only on special indications

⬜ Never

1. **In your Unit, who is responsible for SEDATION level evaluation?**

⬜ Nurse

⬜ Physician

⬜ Trainee

⬜ Nurse practioner

⬜ Other

1. **If you selected “Other” to the previous question, please specify:**
2. **Do you want (optional) to leave your email in order to be acknowkledged at the end of the project?**

_________________________
